# Adulthood weight changes, body mass index in youth, genetic susceptibility and risk of atrial fibrillation: a population-based cohort study

**DOI:** 10.1186/s12916-024-03565-y

**Published:** 2024-08-26

**Authors:** Yufeng Du, Lu Qi, Yan Borné, Emily Sonestedt

**Affiliations:** 1 Department of Epidemiology and Statistics, School of Public Health, Lanzhou University, Lanzhou, Gansu, China; 2 Nutritional Epidemiology, Department of Clinical Sciences Malmö, Lund University, Malmö, Sweden; 3 Department of Epidemiology, School of Public Health and Tropical Medicine, Tulane University, New Orleans, LA USA; 4 Department of Nutrition, Harvard T.H. Chan School of Public Health, Boston, MA USA; 5 Department of Food and Meal Science and the Research Environment MEAL, Faculty of Natural Science, Kristianstad University, Kristianstad, Sweden

**Keywords:** Atrial fibrillation, Body mass index, Weight change, Prospective cohort, Genetic predisposition

## Abstract

**Background:**

Epidemiological evidence on weight change and atrial fibrillation (AF) remains limited and inconsistent. Previous studies on body mass index (BMI) in youth and AF rarely considered subsequent BMI. This study aimed to assess the associations of AF with weight change and BMI in youth, as well as modified effect by genetic susceptibility of AF.

**Methods:**

The study included 21,761 individuals (mean age 57.8 years) from the Malmö Diet and Cancer cohort. Weight information was obtained at three time points, including recalled weight at age 20 years, measured weight at baseline (middle adulthood), and reported weight at 5-year follow-up examination (late middle adulthood). A weighted genetic risk score of AF was created using 134 variants.

**Results:**

During a median follow-up of 23.2 years, a total of 4038 participants developed AF. The association between weight change from early to middle adulthood and AF risk was modified by sex (*P*_interaction_ = 0.004); weight loss was associated with a lower AF risk in females, but not in males. Conversely, weight gain was positively associated with AF risk in a linear manner in females, whereas increased AF risk appeared only when weight gain exceeded a threshold in males. Participants with weight gain of > 5 kg from middle to late middle adulthood had a 19% higher risk of AF relative to those with stable weight, whereas weight loss showed a null association. Compared to individuals with a lower BMI at age 20 years, those with a BMI above 25 kg/m^2^ had an increased risk of AF (HR = 1.14; 95% CI: 1.02–1.28), after controlling for baseline BMI; this association was more pronounced in males or those with a lower genetic risk of AF.

**Conclusions:**

Weight gain in middle adulthood was associated with higher AF risk. Weight loss from early to middle adulthood, but not from middle to late middle adulthood, was associated with a lower risk of AF only in females. Higher BMI in youth was associated with an increased risk of AF, particularly among males or those with a lower genetic risk of AF.

**Supplementary Information:**

The online version contains supplementary material available at 10.1186/s12916-024-03565-y.

## Background

According to the Global Burden of Disease 2019 study, the worldwide prevalent cases of atrial fibrillation (AF) is 59.7 million; almost doubled since 1990 [1]. Approximately 25% of people aged over 55 years in Europe and the USA will develop AF during their later lifetime [2, 3]. Patients with AF are at substantial risk for stroke, heart failure, and all-cause death [4]. Obesity, which has tripled over the past 40 years and affected 14.0% of the world’s adults in 2019 [5], ranks the second largest population-attributable factor for the development of AF following hypertension [6].

The increased risk of AF related to obesity has been well studied in observational and Mendelian randomization studies [7, 8], with the possible mechanisms involving atrial enlargement, conduction abnormalities, and increased atrial fibrosis [9, 10]. However, limited and mixed data exists on weight change and risk of AF [11–16]. Due to limited data and high heterogeneity, the most recent meta-analysis provided only tentative conclusions on weight change and AF risk [17], indicating a positive association between weight gain and AF, while a null association was found with weight loss [17]. Two subsequent cohort studies in Europe, with a relatively small sample size [16] or only covering weight change in late middle adulthood [13], reported a positive association between weight gain and AF but inconsistent results for weight loss [13, 16].

Regarding BMI in youth, five previous studies in Europe, utilizing register-based weight data, consistently showed an elevated risk of AF associated with higher BMI [18–22]. However, previous evidence was limited to either males or females only and did not adjust for several important confounders, such as physical activity, diet, smoking, and alcohol consumption. In previous studies, the median age of participants at the end of follow-up was below 55 years. However, the incidence of AF increases sharply after 65 years old, and more than 70% of prevalent cases are aged 65 years or older [23, 24]. These studies thus missed the opportunity to explore the association of BMI in youth with AF occurred later in life. In addition, in these studies, weight was measured at a single time point, making it unlikely to account for the residual confounding from subsequent BMI. Furthermore, whether genetic susceptibility of AF modified the association of AF with weight change and BMI in youth has not been investigated.

We aimed to investigate the association of weight change from age 20 years to baseline (reflecting weight change from early to middle adulthood), weight change from baseline to follow-up examination (reflecting weight change from middle to late middle adulthood), and BMI at age 20 years with later-life incident AF in the Malmö Diet and Cancer (MDC) cohort, a large population-based prospective cohort with over 23 years of follow-up. We also sought to assess the potential modified effect of the genetic risk score (GRS) of AF on the associations between exposures and AF risk.

## Methods

### Study population

Details of MDC have been published elsewhere [25, 26]. In brief, MDC is a population-based prospective cohort conducted in Malmö, Sweden. Of the 74,318 invited persons, 30,446 individuals aged 45 to 73 years participated in the baseline examination from 1991 to 1996. Participants visited the study center twice at baseline. During the first visit, self-administered questionnaires assessing lifestyle, sociodemographic, and dietary information were explained and distributed to participants. Physical examinations and blood sample collection were performed by trained nurses. Two weeks later, the returned questionnaires were reviewed and a diet interview was performed. About 5 years after baseline, all baseline participants were invited to participate in the follow-up examination. This examination, which collected information on lifestyle, diet, and weight, was conducted between 1997 and 2001. The study was approved by the Ethical Committee at the Medical Faculty at Lund University (approval number: LU 51/90, LU 2009/633, LU 2011/537 and LU 2012/762). All participants provided written informed consent.

A total of 28,098 participants completed the baseline questionnaire, anthropometric measurements, and dietary assessment. For the analyses of BMI at age 20 years and weight change from age 20 years to baseline, we excluded participants with prevalent cancer (*n* = 1747), cardiovascular disease (CVD, *n* = 760), AF (*n* = 223) at baseline, or with missing data on weight aged 20 years (*n* = 3399) or weight at baseline (*n* = 26) or 5 covariates (*n* = 182), finally leaving 21,761 participants for the analyses. For weight change from baseline to follow-up examination, participants who did not attend the follow-up examination (*n* = 5049, with 705 participants due to death), or had incident cancer (*n* = 781), CVD (*n* = 221), or AF (*n* = 173) from baseline to 5-year follow-up examination, or had missing data on weight at baseline (*n* = 22), weight at follow-up examination (*n* = 262), or 4 covariates (*n* = 82), were excluded, leaving 18,778 participants for this analyses. In the analyses involving genetic data, non-European individuals (*n* = 899/*n* = 742) were further excluded. More details are presented in the flowchart (Additional file 1: Fig. S1).

### Exposure assessment

At baseline examination, weight was measured to the nearest 0.1 kg using a balance-beam scale in wearing light indoor clothing and no shoes. Height (cm) was measured in a standing position using a wall-mounted stadiometer. Weight at age 20 years was recalled by participants at baseline. Weight change from age 20 years to baseline (exposure 1) was calculated as baseline weight minus weight at age 20 years and was further divided into the following five groups based on previous studies with similar time length for weight change [27, 28]: loss > 2.5 kg, stable (− 2.5–2.4 kg), gain (2.5–9.9 kg), gain (10–19.9 kg), gain (≥ 20 kg). We chose to use weight change (rather than BMI change) as exposure because absolute weight change can be explained more directly by healthcare providers and policymakers and is easily understood in weight management programs [29]. BMI at age 20 years (exposure 2) was calculated as the recalled weight (kg) divided by the squared height (m) measured at baseline. BMI at age 20 years was further categorized into the following groups [29]: < 18.5 kg/m^2^, 18.5 to 22.4 kg/m^2^, 22.5 to 24.9 kg/m^2^, and ≥ 25 kg/m^2^; overweight and obese participants were categorized into one group due to the small number of obese youth (*n* = 132).

At the 5-year follow-up examination, participants were asked to report their current weight. Weight change from baseline to follow-up examination (exposure 3) was calculated as weight at follow-up examination minus weight at baseline and was further divided into the following five groups based on our data distribution: loss > 5 kg, loss (− 5 to − 2.4 kg), stable (− 2.5–2.4 kg), gain (2.5–4.9 kg), gain (≥ 5 kg). The cutoffs for the extreme weight loss (> 5 kg) and gain (≥ 5 kg) were the 10th and 90th percentiles, respectively. Participants were asked to recall their weight at age 20 years again at follow-up examination, and this variable was used to assess the reliability of the recalled weight at baseline by calculating the intraclass correlation coefficient (ICC).

### AF ascertainment

The outcome AF includes AF and/or atrial flutter (AFL) given their close relationship and the same Swedish ICD-10 coding before January 2013 [19, 30]. Prevalent or incident AF cases were ascertained through the Swedish Hospital Discharge Register and the Swedish Causes of Death Register [31]. The AF cases were defined according to the following codes of the International Classification of Diseases (ICD): 427.92 (ICD-8, used before 1986), 427D (ICD-9, used from 1987 to 1996), and I48 (ICD-10, used since 1997) [31]. In a validation study conducted at the MDC, where AF cases were reviewed by an arrhythmologist and a physician, the registers demonstrated high validity in diagnosing AF, with a positive predictive value of 97% [31]. Follow-up time was calculated from the date of completion of the baseline (for exposure 1 and exposure 2) or of the 5-year follow-up examination (for exposure 3) until the date of the first diagnosis of AF, death, emigration, or 31 December 2018, whichever occurred first. The rate of loss to follow-up due to emigration in this study was less than 1%.

### AF genetic risk score construction

Participants were genotyped using the Illumina GSA v1 genotyping array. The Haplotype Reference Consortium reference was used for imputation [32]. Quality control procedures for the genetic data were described in detail elsewhere [33]. Using 134 single nucleotide polymorphisms (SNPs) identified from a recent meta-analysis of genome-wide association study (GWAS) in Europeans [34], we created a weighted GRS for AF using the following equation: GRS = (β1 × SNP1 + … + β134 × SNP 134) × (134/sum of the β coefficients), with SNP representing risk allele count (0, 1, or 2) for each SNP and β coefficients are weights obtained from GWAS [34]. Low (quintile 1), intermediate (quintiles 2–4), and high (quintile 5) genetic risk categories were created based on the distribution of GRS. We checked the overlapping between 134 SNPs of AF and 941 SNPs of BMI identified in a most recent GWAS in Europeans [35], and no overlapping between the two groups of SNPs was observed.

### Assessment of covariates and other variables

Age and sex were collected from the Swedish registry using a personal identification number. The baseline and follow-up examination took more than 5 years but the follow-up ended at a fixed date, so the year of participating in the baseline or follow-up examination (quartiles) was adjusted in this study. Data on smoking status (current, former, and never), and educational level (elementary, primary and secondary, upper secondary, further education without a degree, and university degree) were collected by a self-administered questionnaire at baseline. Data on alcohol consumption was collected from a food frequency questionnaire (FFQ) and food diary and was further divided into a variable with six categories (zero intake, and sex-specific quintiles for those who reported drinking). The leisure time physical activity, based on 17 different leisure-time physical activities, was categorized into five groups (< 7.5, 7.5–15, 15–25, 25–50, and > 50 MET-h/week). The dietary information was assessed using a modified diet history method including a 7-day food diary, a 168-item semiquantitative FFQ, and a 45–60-min dietary interview. The diet quality index was created by summing up five dietary factors based on the Swedish dietary guidelines: fiber (> 2.4 g/MJ), fruit and vegetables (> 400 g/day), fish (> 300 g/week), added sugar (< 10% energy), and red and processed meat (< 500 g/week) [36]. We assigned a value of 1 to each dietary factor that met the above criteria and 0 otherwise, resulting in a total score between 0 and 5. Participants were asked to rate their health status by the question: how do you feel right now, physically and mentally, with respect to your health and your well-being? The answer was a score ranging from 1 to 7, with a higher score indicating a better health status; a score of 1 refers to “feel very bad, could not feel worse” and 7 refers to “feel very well, could not feel better.”

After participants rested for 10 min, blood pressure was measured by nurses in the supine position using a mercury sphygmomanometer. Hypertension was defined as systolic blood pressure ≥ 140 mmHg and/or diastolic blood pressure ≥ 90 mmHg or the use of antihypertensive medication. Diabetes was ascertained by self-reported diabetes diagnosis or use of diabetes medications or information from the registries mentioned above. The prevalent or incident cases of cancer and CVD were identified by the registries. Information on the use of lipid-lowering medication and prevalent goiter was self-reported. Data on smoking status, alcohol consumption, leisure-time physical activity, self-rated health score, use of lipid-lowering medication, and prevalence of diabetes, hypertension, and goiter were updated during a 5-year follow-up examination.

The variables described below are used in sensitive analysis. The deaths were identified through the Swedish National Tax Agency, Statistics in Sweden, and the National Board of Health and Welfare. Sleep data were collected only during the early and middle periods of the MDC baseline (1991 to 1994), involving 17,537 participants. Data were collected on sleep duration (categorized as 7–8 h or other), waking problems at night (none, small, moderate, very big), and waking up too early problems (none, small, moderate, very big).

### Statistical analyses

Baseline characteristics were presented by exposure categories with means and standard deviations (SD) or percentages. The Cox proportional hazards regression models estimate the associations between exposures and AF risk. The covariates were progressively adjusted in four models. Sex, age, height, and years of participant recruitment were adjusted in model 1. Model 2 was adjusted for variables in model 1 plus educational level, leisure time physical activity, alcohol consumption, smoking status, diet quality index, and self-rated health score. We adjusted for variables in model 2 plus goiter, diabetes, hypertension, lipid-lowering medication (model 3), and further for BMI at age 20 years (continuous, for exposure 1) or BMI at baseline (continuous, for exposure 2 and exposure 3) in model 4 to observe the associations independent of these two variables. For exposure 3, covariates obtained at the follow-up examination were adjusted. All covariates were adjusted in the forms as described in the Covariates Assessment section unless stated otherwise. The proportional hazard assumption was tested by the Schoenfeld test and visual inspection of the Schoenfeld residuals plot. No violation of the assumption was found. To investigate dose–response associations between exposures and AF risk, we fitted restricted cubic spline models with 3 knots. The p-value for nonlinearity was determined from the Wald test. We conducted stratified analyses by sex, age, BMI, smoking status, drinking status, physical activity, diet quality (< 2/ ≥ 2), self-rated health status (< 6/ ≥ 6), GRS, diabetes, hypertension, goiter, and use of lipid-lowering medication to assess potential interactions. P for interaction was determined by Wald tests for cross-product terms (spline basis of exposure × stratification variables). Pearson correlation coefficients between BMI and weight changes were calculated.

The following sensitivity analyses were performed: (1) To minimize the chance of reverse causation, main models were repeated after excluding cases of AF occurring within the first 5 years (*n* = 254 for exposure 1 and exposure 2/*n* = 472 for exposure 3; the numbers below correspond to this pattern) or the first 10 years (*n* = 824/*n* = 1299) of the follow-up; (2) Fine and Grey model was used to account for the competing risks from deaths; (3) given that cancer accounted for one-third of unintentional weight loss cases in older adults, and mental disorders were also linked to such weight loss [37], we repeated the main analyses after excluding participants with low ratings (1 and 2) for physical and mental health (*n* = 575/*n* = 406), self-reported mental disorders (*n* = 179/*n* = 234), or incidents of cancer (*n* = 1120/*n* = 1311) within the first 5 years of follow-up. (4) Since unintentional weight loss can be an early precursor to the occurrence of various life-shortening diseases and has been related to the increased mortality risk [37, 38], we repeated the main analyses after excluding deaths occurring within the first 10 years (*n* = 1526/*n* = 1967) of the follow-up to minimize the confounding from comorbidity-related unintentional weight loss. (5) We adjusted for the three above-mentioned variables of sleep in a subpopulation with available weight and sleep data (*n* = 12,440/*n* = 11,945) because sleep pattern has been reported as a risk factor for AF [39].

Statistical analyses were performed using R version 4.2.1 (R Foundation). Two-sided *P* < 0.05 was considered significant.

## Results

Table 1 presents the baseline characteristics across categories of weight change from age 20 years to baseline. The average weight change was 12.5 kg from age 20 years to baseline. Compared with participants with stable weight, those who were in the highest weight gain group (≥ 20 kg) were more likely to have lower BMI at age 20 years, education levels, alcohol consumption, physical activity, dietary quality index, and self-rated health status. They were also more likely to be former smokers, to use lipid-lowering medication, and to have a higher prevalence of diabetes, hypertension, and goiter at baseline. We observed similar distributions for most characteristics according to weight change from baseline to follow-up examination (Additional file 1: Table S1). Of note, participants who experienced the highest weight loss tended to have higher BMI at baseline, physical activity level, and dietary quality, be current smokers, and have a higher prevalence of diabetes and hypertension (Additional file 1: Table S1). Participants who had a higher BMI (≥ 25 kg/m^2^) at age 20 years tended to be males or former smokers and had a higher prevalence of hypertension and diabetes at baseline relative to those with a lower BMI (18.5–22.4 kg/m^2^) (Additional file 1: Table S2). Weight changes were negatively correlated with the BMIs at the onset of calculating change (Additional file 1: Table S3). A total of 17,427 participants recalled their weight at age 20 years both at baseline and follow-up examination, and ICC was 0.924, indicating high reliability for this exposure.

**Table 1:** Baseline characteristics of study participants according to weight change from age 20 years to baseline

Characteristics	Total	**Absolute weight change from age 20 years to baseline**				
		< − 2.5 kg	− 2.5 to 2.4 kg	2.5 to 9.9 kg	10.0 to 19.9 kg	≥ 20 kg
Participants, *n*	21,761	1072	2032	5696	8219	4742
Age at baseline, years	57.75 ± 7.68	59.41 ± 7.90	57.98 ± 7.92	57.20 ± 7.73	57.50 ± 7.61	58.38 ± 7.50
Female	13,366 (61.4)	684 (63.8)	1213 (59.7)	3586 (63.0)	5047 (61.4)	2836 (59.8)
BMI at age 20 years, kg/m^2^	21.27 ± 2.58	23.97 ± 3.42	22.07 ± 2.42	21.35 ± 2.27	20.97 ± 2.35	20.73 ± 2.69
BMI at baseline, kg/m^2^	25.69 ± 3.95	21.42 ± 2.89	22.19 ± 2.41	23.57 ± 2.31	25.95 ± 2.53	30.25 ± 3.76
Height, cm	168.63 ± 8.84	167.54 ± 8.81	168.36 ± 8.80	168.21 ± 8.62	168.54 ± 8.75	169.65 ± 9.19
University degree	3213 (14.8)	139 (13.0)	336 (16.5)	966 (17.0)	1212 (14.8)	560 (11.8)
Smoking status						
Current	6301 (29.0)	524 (48.9)	786 (38.7)	1766 (31.0)	2142 (26.1)	1083 (22.8)
Former	7276 (33.4)	242 (22.6)	512 (25.2)	1715 (30.1)	2982 (36.3)	1825 (38.5)
Never	8184 (37.6)	306 (28.5)	734 (36.1)	2215 (38.9)	3095 (37.7)	1834 (38.7)
Zero-consumers of alcohol	1295 (6.0)	96 (9.0)	122 (6.0)	255 (4.5)	450 (5.5)	372 (7.8)
High leisure-time physical activity (> 50 MET-h/week)	3649 (16.8)	211 (19.7)	365 (18.0)	1023 (18.0)	1364 (16.6)	686 (14.5)
Diabetes	905 (4.2)	46 (4.3)	60 (3.0)	144 (2.5)	292 (3.6)	363 (7.7)
Hypertension	13,025 (59.9)	544 (50.8)	1029 (50.6)	2962 (52.0)	5002 (60.9)	3488 (73.6)
Goiter	1263 (5.8)	75 (7.0)	105 (5.2)	304 (5.3)	445 (5.4)	334 (7.0)
Lipid-lowering medication	528 (2.4)	11 (1.0)	40 (2.0)	92 (1.6)	217 (2.6)	168 (3.5)
Self-rated health score	5.25 ± 1.31	5.20 ± 1.37	5.42 ± 1.26	5.36 ± 1.25	5.26 ± 1.27	5.04 ± 1.40
Diet quality index	1.90 ± 1.28	1.85 ± 1.32	1.96 ± 1.32	1.93 ± 1.30	1.89 ± 1.26	1.87 ± 1.26

Variables are presented as mean ± SD or *n* (%)

Participants were followed for a median of 23.2 years from baseline and 18.5 years from the follow-up examination, with 4038 and 3547 incident AF cases documented, respectively. The mean age at the diagnosis of AF was 76.7 years (SD, 8.1 years). Table 2 presents the associations between exposures and AF risk. In model 4, the hazard ratio (HR) and 95% CI for participants gaining 5–10 kg and larger than 20 kg weight from age 20 years to baseline were 1.21 (1.07–1.37) and 1.52 (1.34–1.73), respectively, when compared to those with a stable weight. Weight loss over 2.5 kg in this stage was not associated with AF risk. Those who gained weight over 5 kg from baseline to follow-up examination had an elevated AF risk relative to those with stable weight (HR = 1.19; 95% CI, 1.06–1.33). Elevated risk was also seen for those who had a weight loss of over 5 kg in model 3 (HR = 1.25; 95% CI, 1.11–1.40), but this association disappeared after further adjustment for BMI at baseline. Compared with those with a lower BMI at age 20 years (18.5 to 22.4 kg/m^2^), individuals with a higher BMI (≥ 25 kg/m^2^) had a 14% increased risk of AF in Model 4. Adjustment for baseline BMI in Model 4 markedly attenuated the associations observed in Model 3.

**Table 2:** Hazard ratios (95% CIs) for AF by absolute weight change and BMI at age 20 years

	Number of participants	Cases/person years	Model 1	Model 2	Model 3	Model 4
**Weight change from age 20 years to baseline**						
< − 2.5 kg	1072	176/19,818	1.12 (0.93–1.35)	1.09 (0.91–1.31)	1.10 (0.92–1.33)	0.99 (0.82–1.19)
− 2.5 to 2.4 kg	2032	317/40,529	1.00	1.00	1.00	1.00
2.5 to 9.9 kg	5696	895/117,502	1.00 (0.88–1.14)	1.01 (0.89–1.15)	1.00 (0.88–1.13)	1.04 (0.91–1.18)
10 to 19.9 kg	8219	1521/167,571	1.18 (1.05–1.33)	1.18 (1.04–1.33)	1.13 (1.00–1.27)	1.21 (1.07–1.37)
≥ 20 kg	4742	1129/91,618	1.52 (1.34–1.72)	1.50 (1.33–1.71)	1.38 (1.21–1.56)	1.52 (1.34–1.73)
**Weight change from baseline to follow-up examination**						
< − 5 kg	1643	373/24,346	1.35 (1.21–1.51)	1.32 (1.18–1.47)	1.25 (1.11–1.40)	1.04 (0.92–1.17)
− 5 to − 2.4 kg	2751	572/42,970	1.09 (0.99–1.20)	1.09 (0.99–1.19)	1.06 (0.96–1.16)	0.98 (0.89–1.08)
− 2.5 to 2.4 kg	9900	1805/163,880	1.00	1.00	1.00	1.00
2.5 to 4.9 kg	2292	400/38,827	1.03 (0.93–1.15)	1.02 (0.92–1.14)	1.03 (0.92–1.15)	1.04 (0.93––1.16)
≥ 5 kg	2192	397/36,149	1.24 (1.11–1.39)	1.18 (1.06–1.32)	1.19 (1.06–1.33)	1.19 (1.06–1.33)
**BMI at age 20 years (kg/m**^**2**^**)**						
< 18.5	2647	371/55,635	0.94 (0.84–1.05)	0.94 (0.84–1.05)	0.93 (0.83–1.04)	0.99 (0.88–1.10)
18.5 to 22.4	12,966	2318/264,506	1.00	1.00	1.00	1.00
22.5 to 24.9	4612	968/88,827	1.09 (1.01–1.17)	1.09 (1.01–1.17)	1.08 (1.00–1.17)	0.97 (0.90–1.05)
≥ 25	1536	381/28,070	1.49 (1.33–1.66)	1.46 (1.31–1.63)	1.41 (1.27–1.58)	1.14 (1.02–1.28)

Model 1 age, sex, height, and year of participant recruitment. Model 2 covariates in model 1 plus physical activity, alcohol, smoking, educational level, diet quality index, and self-rated health score. Model 3 covariates in model 2 plus goiter, diabetes, hypertension, and lipid-lowering medication. Model 4 covariates in model 3 plus BMI at 20 years old (continuous) for weight change from age 20 years to baseline, and baseline BMI (continuous) for weight change from baseline to 5-year follow-up and BMI at age 20 years

**Fig. 1:**
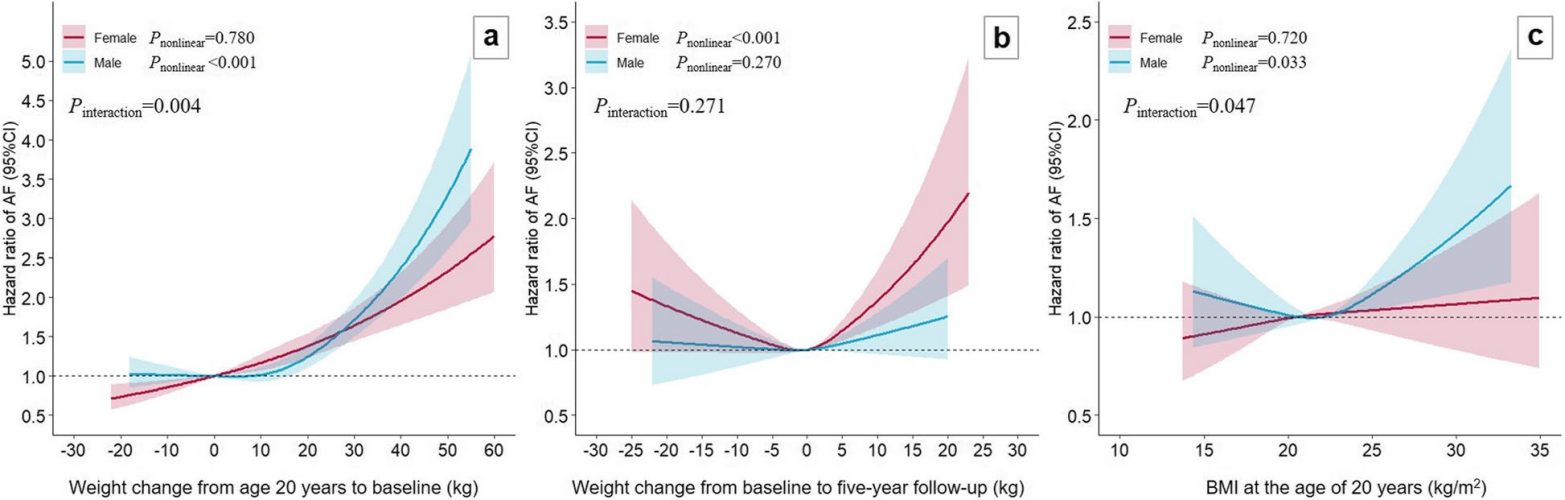
Dose–response associations of AF with weight change from age 20 years to baseline (**a**), weight change from baseline to 5-year follow-up examination (**b**), and BMI at age 20 years (**c**) stratified by sex. The zero (weight change) or median of BMI at age 20 years was used as reference level (HR = 1). Multivariable model adjusted for age, sex, height, year of participants recruitment, physical activity, alcohol, smoking, educational level, diet quality index, self-rated health score, goiter, diabetes, hypertension, lipid-lowering medication, and BMI at age 20 years (continuous) for weight change from age 20 years to baseline, and baseline BMI (continuous) for weight change from baseline to 5-year follow-up examination and BMI at age 20 years

The spline analysis showed significant nonlinear associations of weight change with AF risk (*P*_nonlinear_ < 0.05) (Additional file 1: Fig. S2). Association between weight change from age 20 years to baseline and AF varied by sex (*P*_interaction_ = 0.004): in females, weight loss was associated with a lower AF risk while weight gain was associated with higher risk; in males, AF risk sharply increased with weight gain over approximately 16.5 kg but showed no association when below this threshold (Fig. 1). The association was U-shaped for AF and weight change from baseline to follow-up examination and was not modified by sex (Fig. 1 and Additional file 1: Fig. S2). Sex modified the association between BMI at age 20 years with risk increase associated with higher BMI was only restricted to males (*P*_interaction_ = 0.047) (Fig. 1).

**Fig. 2:**
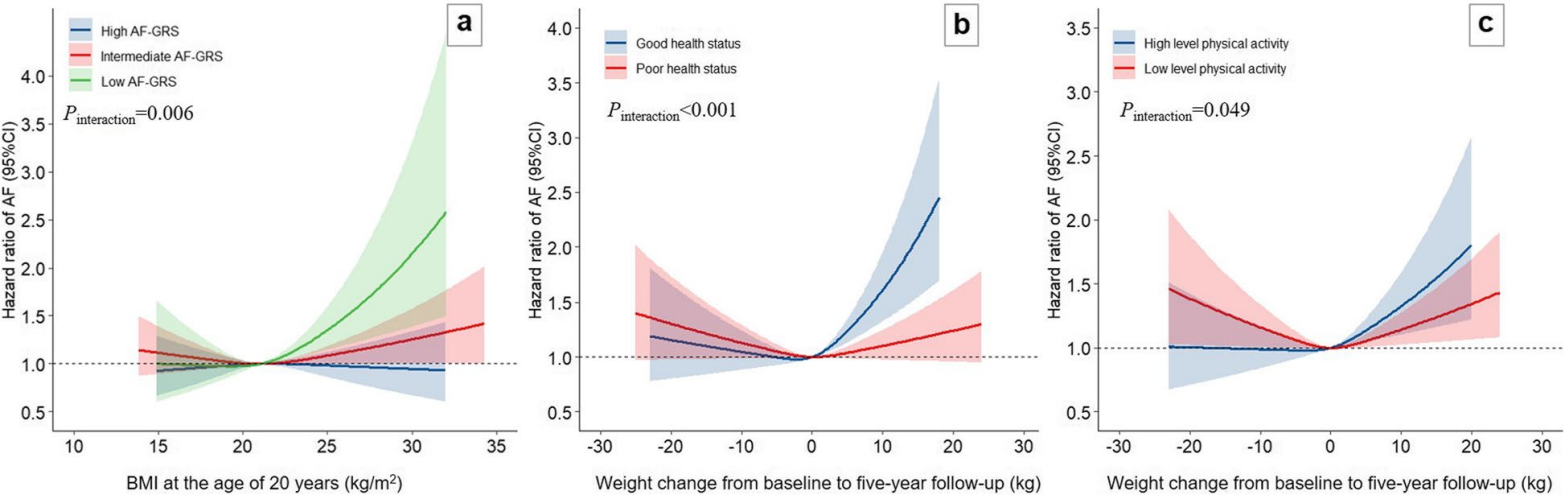
Dose–response associations of AF with BMI at age 20 years (**a**, stratified by GRS), and weight change from baseline to 5-year follow-up examination (**b**, stratified by self-rated health status; **c**, stratified by physical activity). The zero (weight change) or median of BMI at age 20 years was used as reference level (HR = 1). Multivariable model adjusted for age, sex, height, year of participant recruitment, physical activity, alcohol, smoking, educational level, diet quality index, self-rated health score, goiter, diabetes, hypertension, and lipid-lowering medication, baseline BMI (continuous)

In stratified analyses by age, BMI, drinking status, diet quality, diabetes, hypertension, goiter, and use of lipid-lowering medication, the results were similar in each stratum (Additional file 1: Table S4). However, a modified effect was observed for self-rated health status, physical activity, and GRS of AF. We presented the results with significant interactions in Fig. 2. The increased AF risk associated with higher BMI at age 20 years tended to be restricted to individuals who had a lower genetic risk of AF (*P*_interaction_ = 0.006). For weight change from baseline to follow-up examination, an elevated risk of AF associated with weight loss was observed for those who had a lower physical activity level (*P*_interaction_ = 0.049), and higher AF risk with weight gain appears to be stronger in individuals with better health status (*P*_interaction_ < 0.001). Smoking showed a borderline interaction with weight change from baseline to follow-up examination (*P*_interaction_ = 0.067) (Additional file 1: Fig. S3).

The results for sensitivity analyses are presented in Additional file 1: Tables S5–S7. The results were similar after excluding AF cases occurring within the first 5 or 10 years of follow-up, excluding participants with a poor health condition, mental disorder, and incidents of cancer within the first 5 years of the follow-up, or deaths occurring within the first 10 years of the follow-up, although the magnitude of some associations was slightly changed. In a subpopulation with available sleep data, additional adjustments for sleep variables yielded similar results. When using the percentage of weight change as the exposure, similar results were observed (Additional file 1: Fig. S4).

## Discussion

In this large population, we found that the association between weight change from early to middle adulthood and AF risk varied by sex; weight loss was associated with a lower AF risk only in females, and a positive association with weight gain showed a linear pattern in females but became significant only when weight gain exceeded a threshold in males. Weight gain from middle to late middle adulthood was associated with higher AF risks, whereas weight loss showed no significant association. The genetic susceptibility of AF did not modify findings of weight change. Having a higher BMI in youth was associated with an increased risk of AF; this association was modified by sex and GRS of AF. Main results were robust in sensitivity analyses and most subgroups.

Two previous studies on weight gain from early to middle adulthood and AF risk reported similar positive associations as in our study [11, 16]. The HUNT study, which included 15,214 individuals, showed that a BMI gain of more than 5 kg/m^2^ was associated with a 2.6-fold increase in the risk of AF [16]. Another Swedish cohort (including 6903 males) found a risk increase of 90% for a weight gain of more than 35% versus those with a stable weight [11]. For weight gain from middle to late middle adulthood, our findings were supported by two cohorts in Denmark [13] and Sweden [15]. The Atherosclerosis Risk in Communities (ARIC) study found an increase in AF risk associated with weight gain in males but not in females [14], while the Danish cohort [13] and our study demonstrated that this association was not modified by sex. This inconsistency is possibly due to statistical power, as the Danish cohort and our study had a larger number of AF cases than the ARIC study (*n* > 4000 versus *n* = 1775). Our study adds new knowledge that the association between weight change and AF was not modified by the genetic risk of AF, which suggests that all individuals, regardless of their genetic susceptibility to AF, would benefit from avoiding substantial weight gain.

Weight gain from early to late middle adulthood mainly reflects the accumulation of fat mass, especially visceral adipose tissue [40, 41]. Epicardial fat plays a crucial role in AF pathogenesis, potentially via promoting fibrosis, triggering electrical remodeling, and inducing proinflammatory, profibrotic, and proarrhythmic effects [9]. Moreover, the effect of weight gain on AF risk may be mediated through the increases in cardiometabolic diseases, such as hypertension, diabetes, and obstructive sleep apnea [42], all of which are involved in the pathogenesis of AF.

In females, we noted an inverse association between weight loss from early to middle adulthood and AF. However, the HUNT study showed null associations [16]. This study had a smaller sample size than ours. Additionally, the benefits of weight loss may be masked by a higher initial BMI, considering that weight change was inversely associated with BMI at age 20 years (*r* =  − 0.23), a finding also observed in a large US cohort (*r* =  − 0.24) [28]. However, this adjustment was not performed in the HUNT study. Our observed null association between weight loss and AF in males was strengthened by a Swedish cohort comprising only male participants [11].

Previous studies on weight loss in middle or late middle adulthood produced mixed results, including positive [14], negative [12], and null associations [13]. The Danish cohort [13], in which baseline BMI was adjusted, showed a similar null association as in our fully adjusted models. The ARIC study [14] observed a positive association between weight loss and AF risk, which is similar to our results in models 1–3. This association, however, attenuated markedly and became nonsignificant after controlling for baseline BMI. This adjustment was not performed in the ARIC study. Weight change is negatively correlated with baseline BMI (*r* =  − 0.25) in our study, which suggests that this elevated AF risk was mainly contributed by higher baseline BMI rather than the weight loss itself. By contrast, a study in Israel, using screened data from a tertiary medical center, saw a risk reduction of 12% for a 5-kg weight loss [12]. This inconsistency with ours and the Danish cohort may result from the younger age of their participants (49 versus 58/61 years). An explanation is that, in younger participants, who are less susceptible to chronic conditions causing unintentional weight loss, the association of AF with weight loss is, therefore, less likely to be confounded.

In stratified analysis, an increased risk of AF associated with weight loss from middle to late middle adulthood tends to be limited to individuals with low levels of physical activity or poor health status, or to former and current smokers. These results collectively support the likelihood that this increased AF risk among them may be due to confounding from underlying comorbidities related to unintentional weight loss. Nevertheless, in individuals with good health status, assumed to be less prone to comorbidities, weight loss was not associated with lower AF risk. Moreover, in sensitivity analysis, individuals with low ratings for their mental and physical health, incidents of cancer within the first 5 years of follow-up, as well as deaths occurring within the first 10 years of follow-up, were excluded to minimize confounding from underlying comorbidities. Weight loss remained unrelated to lower AF risk in these analyses. Based on these considerations and previous evidence [17], another explanation that obesity-induced changes in cardiac structure and function are less likely to be reversible via weight loss occurring in late middle adulthood seems more plausible [43]. Despite both fat-free and fat body mass have been shown to be causally related to a higher risk of AF [44], our study added the evidence that weight loss occurring in the later stage of middle adulthood is not associated with a reduced risk of AF. More large cohorts covering early and middle adulthood, with multiple weight assessments, are needed to explore more specific stages when the risk of AF can be reversed by weight loss. The findings regarding weight change and AF risk may not be directly applicable to the secondary prevention of AF; this is due to the observation of the “obesity paradox” phenomenon in AF, suggesting that obesity may confer benefits for those with prevalent AF [45–47].

Our findings for BMI at age 20 years corroborate previous studies using data from military conscripts or medical birth registries [18–22]. We extended previous evidence by accounting for subsequent BMI, suggesting that higher BMI in youth is a crucial risk factor for AF irrespective of their subsequent BMI. The mechanisms underlying this finding may involve obesity-related unfavorable changes in metabolism and myocardial structure and function, which have been noted in adolescents [48]. Our observed increased risk related to a higher BMI was limited to males. This is plausible as more apparent obesity-related alterations in cardiometabolic risk factors have been noted in boys and young men [49]. Another finding was that increased risk in relation to a higher BMI seems more relevant in individuals with a lower genetic risk of AF; this novel finding warrants confirmation in further studies. It is worth noting that BMI is less sensitive in identifying excess adiposity in youth [50]. Using other measures, such as body fat percentage, may provide new insights.

This study has several strengths. To our knowledge, this study is the first to account for genetic risk and subsequent BMI in the analysis of BMI in youth. The large sample size and long follow-up period of this study ensured the ability to conduct multiple stratified and sensitivity analyses, assess long-term risk, and capture the periods of high AF incidence. Other strengths are its population-based prospective design and coverage of two time periods of weight change.

Some limitations of this study also need to be noted. First, residual confounding is unavoidable due to the observational nature of our study despite the most identified risk factors were carefully considered. Second, recall bias was inevitable for exposure weight at age 20 years. However, our data indicated the high reliability of this exposure. A meta-analysis of validation studies revealed a correlation coefficient of 0.82 between recalled early-life weight and measured weight, with a small mean difference of 0.87 kg [51]. In addition, the distribution of BMI at age 20 years in our study is similar to that in a study using measured data from Swedish military conscripts during 1969–1997 (including 1,547,478 men aged 18 years) [20]. The proportions of being normal, overweight, and obese were 93.7%, 5.4%, and 1.0% in our male participants, and 92.2%, 5.5%, and 2.3% in their study, respectively. Third, we use height measured at baseline in calculating BMI at age 20 years, which could introduce systematic error, especially for those who were older at baseline, since aging is accompanied by a decrease in height [52]. This error may not alter our findings, given similar associations across strata in the stratified analysis by age. Fourth, we have no data on intentional and unintentional weight loss, which is common in observational studies considering the difficulty in diagnosing unintentional weight loss [37]. Fifth, because AF cases were identified through registers in this study, undiagnosed cases, especially asymptomatic ones, may exist, and different patterns of AF (i.e., first-diagnosed AF, paroxysmal AF, and persistent AF), which exhibit distinct impacts on clinical outcomes [53], could not be separated. Further, atrial cardiomyopathy, characterized by electrophysiological, contractile/functional, and structural changes in the atria and playing a pivotal role in the pathology of AF [54], was not feasible in this study. Future studies exploring various patterns of AF and atrial cardiomyopathy could help refine weight management targets for each specific pattern and aid in the development of early prevention strategies for AF. Finally, our study only included participants living in Sweden, and prevalent cancer and CVD cases were excluded to enhance the internal validity, but this limits the generalizability of our findings to other populations.

## Conclusions

Weight gain during middle adulthood contributes to an increased risk of AF, whereas weight loss, occurring only in the early stage of middle adulthood, was associated with a lower AF risk in females. Associations of AF with weight change were similar across all levels of AF genetic risk. Having a higher BMI in youth, particularly in males or those with a low genetic risk of AF, is related to a higher risk of AF in later life, independent of their subsequent BMI. These findings suggest a need to implement weight management in youth and middle adulthood for the primary prevention of AF. Results on interactions with sex and genetic risk may assist future public health programs in identifying target intervention populations.

### Supplementary Information

Additional file 1:  Table S1. Baseline characteristics of study participants according to weight change from baseline to follow-up examination. Table S2. Baseline characteristics of study participants according to BMI at age 20 years. Table S3. Correlation coefficients for BMI at three time points and weight changes during two intervals. Table S4. Assessments of the modified effect by key variables on the association between exposures and AF. Table S5. Sensitivity analyses of the associations between weight change from age 20 years to baseline and risk of AF. Table S6. Sensitivity analyses of the associations between weight change from baseline to follow-up examination and risk of AF. Table S7. Sensitivity analyses of the associations between BMI at age 20 years and risk of AF. Fig. S1. The flowchart for selecting participants. Fig. S2. Dose–response association of AF with weight change and BMI at age 20 years. Fig. S3. Dose–response associations of AF with weight change from baseline to five-year follow-up examination stratified by smoking status. Fig. S4. Dose–response associations of AF with percentage of weight change from age 20 years to baseline (a) and percentage of weight change from baseline to five-year follow-up examination (b) stratified by sex.

## Data Availability

Supporting data are available from the corresponding author upon reasonable request but access to data must be granted by the MDC steering committees.

## Abbreviations

AF: Atrial fibrillation
AFL: Atrial flutter
ARIC: Atherosclerosis risk in communities
BMI: Body mass index
CVD: Cardiovascular disease
FFQ: Food frequency questionnaire
GRS: Genetic risk score
GWAS: Genome-wide association study
HR: Hazard ratio
ICC: Intraclass correlation coefficient
ICD: International classification of diseases
MDC: Malmö Diet and Cancer
SD: Standard deviation
SNP: Single nucleotide polymorphism
