# Supplementary material for: Adulthood weight changes, body mass index in youth, genetic susceptibility and risk of atrial fibrillation: a population-based cohort study
Source: BMC Med. 2024 Aug 26;22:345. doi: 10.1186/s12916-024-03565-y (PMC11346199; doi:10.1186/s12916-024-03565-y)
Supplement: Supplementary file 1 — Additional file 1: Table S1. Baseline characteristics of study participants according to weight change from baseline to follow-up examination. Table S2. Baseline characteristics of study participants according to BMI at age 20 years. Table S3. Correlation coefficients for BMI at three time points and weight changes during two intervals. Table S4. Assessments of the modified effect by key variables on the association between exposures and AF. Table S5. Sensitivity analyses of the associations between weight change from age 20 years to baseline and risk of AF. Table S6. Sensitivity analyses of the associations between weight change from baseline to follow-up examination and risk of AF. Table S7. Sensitivity analyses of the associations between BMI at age 20 years and risk of AF. Fig. S1. The flowchart for selecting participants. Fig. S2. Dose–response association of AF with weight change and BMI at age 20 years. Fig. S3. Dose–response associations of AF with weight change from baseline to five-year follow-up examination stratified by smoking status. Fig. S4. Dose–response associations of AF with percentage of weight change from age 20 years to baseline (a) and percentage of weight change from baseline to five-year follow-up examination (b) stratified by sex. [file 12916_2024_3565_MOESM1_ESM.docx]

**Adulthood weight changes, body mass index in youth, genetic susceptibility and risk of atrial fibrillation: a population-based cohort study**

**Supplemental Content:**

**Table S1**. Baseline characteristics of study participants according to weight change from baseline to follow-up examination.

**Table S2.** Baseline characteristics of study participants according to BMI at age 20 years.

**Table S3.** Correlation coefficients for BMI at three time points and weight changes during two intervals.

**Table S4.** Assessments of the modified effect by key variables on the association between exposures and AF.

**Table S5.** Sensitivity analyses of the associations between weight change from age 20 years to baseline and risk of AF.

**Table S6.** Sensitivity analyses of the associations between weight change from baseline to follow-up examination and risk of AF.

**Table S7.** Sensitivity analyses of the associations between BMI at age 20 years and risk of AF.

**Fig. S1.** The flowchart for selecting participants.

**Fig. S2.** Dose-response association of AF with weight change and BMI at age 20 years.

**Fig. S3.** Dose-response associations of AF with weight change from baseline to five-year follow-up examination stratified by smoking status.

**Fig. S4.** Dose-response associations of AF with percentage of weight change from age 20 years to baseline (a) and percentage of weight change from baseline to five-year follow-up examination (b) stratified by sex.

| **Table S1**. Baseline characteristics of study participants according to weight change from baseline to follow-up examination^a^ | | | | | | | |
| --- | --- | --- | --- | --- | --- | --- | --- |
| Characteristics | Total |  | **Weight change from baseline to five-year follow-up** | | | | |
|  |  |  | < -5kg | -5kg to -2.5kg | -2.5kg to 2.5kg | 2.5kg to 5kg | >= 5kg |
| Participants, n | 18778 |  | 1643 | 2751 | 9900 | 2292 | 2192 |
| Age at baseline, years | 61.96±7.43 |  | 63.18±7.63 | 63.43±7.61 | 62.07±7.44 | 60.80±6.96 | 59.93±6.80 |
| Female | 11651 (62.1) |  | 1046 (63.7) | 1632 (59.3) | 6141 (62.0) | 1424 (62.1) | 1408 (64.2) |
| BMI at baseline, kg/m^2^ | 25.56±3.87 |  | 28.99±4.51 | 26.69±3.69 | 24.96±3.48 | 24.81±3.56 | 25.10±3.89 |
| Height, cm | 168.66±8.81 |  | 169.07±9.05 | 168.97±8.88 | 168.48±8.74 | 168.67±8.85 | 168.75±8.77 |
| University degree | 2802 (14.9) |  | 211 (12.8) | 379 (13.8) | 1592 (16.1) | 325 (14.2) | 295 (13.5) |
| Smoking status |  |  |  |  |  |  |  |
| Current | 4067 (21.7) |  | 396 (24.1) | 630 (22.9) | 2065 (20.9) | 487 (21.3) | 489 (22.3) |
| Former | 7066 (37.6) |  | 617 (37.6) | 963 (35.0) | 3584 (36.2) | 877 (38.3) | 1025 (46.8) |
| Never | 7645 (40.7) |  | 630 (38.3) | 1158 (42.1) | 4251 (42.9) | 928 (40.5) | 678 (30.9) |
| Zero-consumers of alcohol | 1021 (5.4) |  | 121 (7.4) | 159 (5.8) | 469 (4.7) | 118 (5.2) | 154 (7.0) |
| High leisure-time physical  activity (> 50METh/week) | 2818 (15.0) |  | 270 (16.4) | 441 (16.0) | 1508 (15.2) | 332 (14.5) | 267 (12.2) |
| Diabetes | 1140 (6.1) |  | 234 (14.2) | 234 (8.5) | 447 (4.5) | 95 (4.1) | 130 (5.9) |
| Hypertension | 11236 (59.8) |  | 1173 (71.4) | 1820 (66.2) | 5802 (58.6) | 1283 (56.0) | 1158 (52.8) |
| Goiter | 1273 (6.8) |  | 114 (6.9) | 195 (7.1) | 636 (6.4) | 162 (7.1) | 166 (7.6) |
| Lipid-lowering medication | 1178 (6.3) |  | 130 (7.9) | 184 (6.7) | 572 (5.8) | 130 (5.7) | 162 (7.4) |
| Self-rated health score | 5.37±1.28 |  | 5.30±1.40 | 5.44±1.30 | 5.45±1.23 | 5.31±1.25 | 5.01±1.37 |
| Diet quality index | 1.95±1.29 |  | 1.99±1.28 | 1.97±1.28 | 1.97±1.30 | 1.92±1.29 | 1.85±1.27 |
| ^a^Variables are presented as mean± SD or n (%). | | | | | | | |

| **Table S2.** Baseline characteristics of study participants according to BMI at age 20 years^a^ | | | | | | |  |
| --- | --- | --- | --- | --- | --- | --- | --- |
| Characteristics | Total |  | **BMI at age 20 years (kg/m^2^)** | | | | |
|  |  |  | <18.5 | 18.5 to 22.4 | 22.5 to 24.9 | ≥ 25.0 | |
| Participants, n | 21761 |  | 2647 | 12966 | 4612 | 1736 | |
| Age at baseline, years | 57.75±7.68 |  | 55.29±7.55 | 57.53±7.61 | 59.31±7.56 | 59.20±7.56 | |
| Female | 13366 (61.4) |  | 2219 (83.8) | 8449 (65.2) | 1983 (43.0) | 715 (46.6) | |
| BMI at baseline, kg/m^2^ | 25.69±3.95 |  | 23.56±3.33 | 25.08±3.47 | 27.34±3.76 | 29.60±4.88 | |
| Height, cm | 168.63±8.84 |  | 167.23±7.49 | 168.45±8.87 | 169.87±8.97 | 168.90±9.83 | |
| University degree | 3213 (14.8) |  | 406 (15.3) | 2062 (15.9) | 594 (12.9) | 151 (9.8) | |
| Smoking status |  |  |  |  |  |  | |
| Current | 6301 (29.0) |  | 828 (31.3) | 3689 (28.5) | 1312 (28.5) | 472 (30.7) | |
| Former | 7276 (33.4) |  | 798 (30.2) | 4360 (33.6) | 1562 (33.9) | 556 (36.2) | |
| Never | 8184 (37.6) |  | 1021 (38.6) | 4917 (37.9) | 1738 (37.7) | 508 (33.1) | |
| Zero-consumers of alcohol | 1295 (6.0) |  | 169 (6.4) | 717 (5.5) | 282 (6.1) | 127 (8.3) | |
| High leisure-time physical  activity (> 50METh/week) | 3649 (16.8) |  | 414 (15.6) | 2104 (16.2) | 883 (19.2) | 248 (16.2) | |
| Diabetes | 905 (4.2) |  | 91 (3.4) | 467 (3.6) | 217 (4.7) | 130 (8.5) | |
| Hypertension | 13025 (59.9) |  | 1420 (53.7) | 7468 (57.6) | 3037 (65.9) | 1100 (71.6) | |
| Goiter | 1263 (5.8) |  | 189 (7.1) | 790 (6.1) | 211 (4.6) | 73 (4.8) | |
| Lipid-lowering medication | 528 (2.4) |  | 66 (2.5) | 281 (2.2) | 133 (2.9) | 48 (3.1) | |
| Self-rated health score | 5.25±1.31 |  | 5.22±1.28 | 5.26±1.29 | 5.27±1.34 | 5.15±1.36 | |
| Diet quality index | 1.90±1.28 |  | 1.80±1.26 | 1.91±1.28 | 1.91±1.27 | 1.94±1.27 | |
| ^a^Variables are presented as mean± SD or n (%). | | | | | | |  |

| **Table S3.** Correlation coefficients for BMI at three time points and weight changes during two intervals^a^ | | | | | | |
| --- | --- | --- | --- | --- | --- | --- |
|  | **BMI** | | |  | **Absolute weight change** | |
|  | At age 20 years | At baseline | At follow-up examination |  | From age 20 years to baseline | From baseline to follow-up examination |
| BMI at age 20 years | 1.00 | - | - |  | - | - |
| BMI at baseline | 0.43 | 1.00 | - |  | - | - |
| BMI at follow-up examination | 0.41 | 0.90 | 1.00 |  | - | - |
| Weight change from age 20 years to baseline | -0.23 | 0.77 | 0.68 |  | 1.00 | - |
| Weight change from baseline to follow-up examination | -0.08 | -0.26 | 0.17 |  | -0.24 | 1.00 |
| ^a^ All correlation coefficients are significant at the 0.001 level. | | | | | | |

| **Table S4.** Assessments of the modified effect by key variables on the association between exposures and AF^a^ | | | | | |
| --- | --- | --- | --- | --- | --- |
|  | Weight change from age 20 years to baseline (exposure1) |  | Weight change from baseline to follow-up examination  (exposure 3) |  | BMI at age 20 years (exposure 2) |
|  | P value for interaction |  | P value for interaction |  | P value for interaction |
| Age | 0.739 |  | 0.995 |  | 0.219 |
| BMI | 0.139 |  | 0.463 |  | 0.563 |
| GRS of AF | 0.634 |  | 0.374 |  | **0.006** |
| Smoking status | 0.749 |  | **0.067** |  | 0.170 |
| Drinking status | 0.923 |  | 0.380 |  | 0.332 |
| Diet quality | 0.504 |  | 0.249 |  | 0.219 |
| Physical activity | 0.954 |  | **0.049** |  | 0.414 |
| Self-rated health status | 0.360 |  | **<0.001** |  | 0.322 |
| Diabetes | 0.229 |  | 0.148 |  | 0.990 |
| Hypertension | 0.056 |  | 0.897 |  | 0.465 |
| Goiter | 0.327 |  | 0.647 |  | 0.830 |
| Lipid-lowering medication | 0.550 |  | 0.785 |  | 0.301 |
| ^a^ The variable categories used in the stratified analysis are as follows: age at baseline (years: <55, 55-64.9, ≥65) for exposure 1 and exposure 2 or age at follow-up examination (years: <60, 60-69.9, ≥70) for exposure 3, BMI at baseline (kg/m^2^: <25, 25–29.9, ≥ 30) for exposure 2 and exposure 3 or BMI at age 20 years (kg/m^2^: <18.5. 18.5–22.4, ≥ 22.5) for exposure 1, GRS of AF (low, intermediate, and high genetic risk), smoking status (never/former and current), drinking status (sex-specific median: < median/≥median), diet quality (<2/≥2), physical activity (METh/week: <25/≥25), self-rated health status (<6/≥6), diabetes (yes/no), hypertension (yes/no), goiter (yes/no), lipid-lowering medication (yes/no). All variables (except dietary quality) were updated in the analysis of exposure 3. | | | | | |

| **Table S5.** Sensitivity analyses of the associations between weight change from age 20 years to baseline and risk of AF^a^ | | | | | |
| --- | --- | --- | --- | --- | --- |
|  | **Weight change from age 20 years to baseline** | | | | |
|  | < -2.5 kg | -2.5 to 2.5 kg | 2.5 to 10.0 kg | 10 to 20 kg | ≥20 kg |
| Excluding cases of AF occurring within the first 5 years | 0.99 (0.82-1.21) | 1.00 | 1.03 (0.90-1.17) | 1.21 (1.06-1.37) | 1.52 (1.33-1.74) |
| Excluding cases of AF occurring within the first 10 years | 1.05 (0.85-1.30) | 1.00 | 1.09 (0.95-1.27) | 1.26 (1.08-1.43) | 1.54 (1.33-1.78) |
| Competing risk model | 0.92 (0.76-1.12) | 1.00 | 1.10 (0.96-1.25) | 1.28 (1.13-1.45) | 1.57 (1.38-1.79) |
| Excluding participants with poor health conditions, self-reported mental disorders, and incidents of cancer within the first 5 years of follow-up | 0.98 (0.81-1.20) | 1.00 | 1.04 (0.91-1.18) | 1.23 (1.08-1.40) | 1.54 (1.34-1.76) |
| Excluding deaths occurring within the first 10 years of the follow-up | 0.99 (0.82-1.21) | 1.00 | 1.05 (0.92-1.20) | 1.23 (1.08-1.39) | 1.56 (1.37-1.78) |
| Further adjustment for sleep data | 0.91 (0.70-1.17) | 1.00 | 1.07 (0.90-1.26) | 1.17 (1.00-1.38) | 1.54 (1.30-1.83) |
| ^a^Adjusted for age, sex, year of participant recruitment, physical activity, alcohol, smoking, educational level, diet quality index, self-rated health score, goiter, diabetes, hypertension, lipid-lowering medication, and BMI at age 20 years. | | | | | |

| **Table S6.** Sensitivity analyses of the associations between weight change from baseline to follow-up examination and risk of AF^a^ | | | | | |
| --- | --- | --- | --- | --- | --- |
|  | **Weight change from baseline to follow-up examination** | | | | |
|  | < -5kg | -5 to -2.5kg | -2.5 to 2.5kg | 2.5 to 5kg | ≥ 5kg |
| Excluding cases of AF occurring within the first 5 years | 1.02 (0.90-1.17) | 0.99 (0.89-1.10) | 1.00 | 1.04 (0.92-1.17) | 1.25 (1.11-1.40) |
| Excluding cases of AF occurring within the first 10 years | 1.08 (0.93-1.26) | 1.02 (0.91-1.16) | 1.00 | 1.08 (0.94-1.23) | 1.27 (1.11-1.46) |
| Competing risk model | 0.94 (0.83-1.07) | 0.95 (0.86-1.05) | 1.00 | 1.05 (0.94-1.17) | 1.13 (1.01-1.27) |
| Excluding participants with poor health conditions, self-reported mental disorders, and incidents of cancer within the first 5 years of follow-up | 0.99 (0.87-1.12) | 0.97 (0.88-1.07) | 1.00 | 1.02 (0.91-1.14) | 1.19 (1.06-1.33) |
| Excluding deaths occurring within the first 10 years of the follow-up | 1.01 (0.90-1.14) | 0.98 (0.89-1.08) | 1.00 | 1.04 (0.93-1.16) | 1.20 (1.07-1.34) |
| Further adjustment for sleep data | 1.06 (0.90-1.23) | 1.01 (0.89-1.14) | 1.00 | 1.05 (0.93-1.20) | 1.18 (1.04-1.35) |
| ^a^Adjusted for age, sex, year of participant recruitment, physical activity, alcohol, smoking, educational level, diet quality index, self-rated health score, goiter, diabetes, hypertension, and lipid-lowering medication. | | | | | |

| **Table S7.** Sensitivity analyses of the associations between BMI at age 20 years and risk of AF^a^ | | | | |
| --- | --- | --- | --- | --- |
|  | **BMI at age 20 years (kg/m^2^)** | | | |
|  | < 18.5 | 18.5 to 22.4 | 22.5 to 24.9 | ≥ 25 |
| Excluding cases of AF occurring within the first 5 years | 0.97 (0.86-1.08) | 1.00 | 1.00 (0.93-1.09) | 1.21 (1.08-1.37) |
| Excluding cases of AF occurring within the first 10 years | 0.97 (0.86-1.10) | 1.00 | 1.01 (0.92-1.10) | 1.23 (1.08-1.40) |
| Competing risk model | 0.96 (0.86-1.07) | 1.00 | 0.96 (0.88-1.04) | 1.11 (0.99-1.25) |
| Excluding participants with poor health conditions, self-reported mental disorders, and incidents of cancer within the first 5 years of follow-up | 0.99 (0.88-1.11) | 1.00 | 0.99 (0. 91-1.07) | 1.15 (1.02-1.29) |
| Excluding deaths occurring within the first 10 years of the follow-up | 0.99 (0.88-1.11) | 1.00 | 0.97 (0.90-1.05) | 1.14 (1.02-1.29) |
| Further adjustment for sleep data | 1.08 (0.93-1.25) | 1.00 | 0.98 (0.88-1.08) | 1.14 (0.98-1.32) |
| ^a^Adjusted for age, sex, year of participant recruitment, physical activity, alcohol, smoking, educational level, diet quality index, self-rated health score, goiter, diabetes, hypertension, lipid-lowering medication, and BMI at baseline. | | | | |

**Fig. S1** The flowchart for selecting participants.

**Fig. S2** Dose-response association of AF with weight change from age 20 years to baseline (a), weight change from baseline to five-year follow-up survey (b), and BMI at age 20 years (c). The zero (weight change) or median of BMI at age 20 year (21.05 kg/m^2^) was used as reference level (HR=1). Multivariable model adjusted for age, sex, height, year of participant recruitment, physical activity, alcohol, smoking, educational level, diet quality index, and self-rated health score, goitre, diabetes, hypertension, and lipid-lowering medication, BMI at age 20 years (continuous) for weight change from age 20 years to baseline, and baseline BMI (continuous) for weight change from baseline to five-year follow-up examination and BMI at age 20 years.

**Fig. S3** Dose-response associations of AF with weight change from baseline to five-year follow-up examination stratified by smoking status. The zero was used as reference level (HR=1). Multivariable model adjusted for age, sex, height, year of participant recruitment, physical activity, alcohol, educational level, diet quality index, self-rated health score, goiter, diabetes, hypertension, and lipid-lowering medication, baseline BMI (continuous).

**Fig. S4** Dose-response associations of AF with **percentage of weight change** from age 20 years to baseline (a) and **percentage of weight change** from baseline to five-year follow-up examination (b) stratified by sex. The zero was used as reference level (HR=1). Multivariable model adjusted for age, sex, height, year of participants recruitment, physical activity, alcohol, smoking, educational level, diet quality index, self-rated health score, goiter, diabetes, hypertension, lipid-lowering medication, and BMI at age 20 years (continuous) for weight change from age 20 years to baseline, and baseline BMI (continuous) for weight change from baseline to five-year follow-up examination. The percentage of weight change was calculated by subtracting the current weight from the previous weight and then dividing by the previous weight.
